# Comparison of PDMS and NOA Microfluidic Chips: Deformation, Roughness, Hydrophilicity and Flow Performance

**DOI:** 10.3390/mi14112033

**Published:** 2023-10-31

**Authors:** Tatiana Turcitu, Curtis J. K. Armstrong, Niko Lee-Yow, Maya Salame, Andy Vinh Le, Marianne Fenech

**Affiliations:** Department of Mechanical Engineering, University of Ottawa, Ottawa, ON K1N 6N5, Canadamsala029@uottawa.ca (M.S.);

**Keywords:** characteristic time, resistance, compliance, microfluidics, PDMS, NOA 63, contact angle, hydrophilicity, roughness, young modulus, RC hydraulic circuit model

## Abstract

Microfluidic devices are frequently manufactured with polydimethylsiloxane (PDMS) due to its affordability, transparency, and simplicity. However, high-pressure flow through PDMS microfluidic channels lead to an increase in channel size due to the compliance of the material. As a result, longer response times are required to reach steady flow rates, which increases the overall time required to complete experiments when using a syringe pump. Due to its excellent optical properties and increased rigidity, Norland Optical Adhesive (NOA) has been proposed as a promising material candidate for microfluidic fabrication. This study compares the compliance and deformation properties of three different characteristic sized (width of parallel channels: 100, 40 and 20 µm) microfluidic devices made of PDMS and NOA. The comparison of the microfluidics devices is made based on the Young’s modulus, roughness, contact angle, channel width deformation, flow resistance and compliance. The experimental resistance is estimated through the measurement of the flow at a given pressure and a precision flow meter. The characteristic time of the system is extracted by fitting the two-element resistance-compliance (RC) hydraulic circuit model. The compliance of the microfluidics chips is estimated through the measurement of the characteristic time required for channels to achieve an output flow rate equivalent to that of the input flow rate using a syringe pump and a precision flow meter. The Young modulus was found to be 2 MPa for the PDMS and 1743 MPa for the NOA 63. The surface roughness was found to be higher for the NOA 63 than for the PDMS. The hydrophilicities of materials were found comparable with and without plasma treatment. The results show that NOA devices have lower compliance and deformation than PDMS devices.

## 1. Introductions

Microfluidic chips are mostly manufactured using Polydimethylsiloxane (PDMS) [1]. PDMS is widely used due to its transparency [2], low costs, and ease of manufacturing [1,3,4]. The prefusion of microfluidic chips can require hundreds of bars to overcome the system’s resistance. Under pressure, the dimension of the channels can significantly change depending on the compliance [5]. Constant and precise dimensions of microfluidic channels are crucial for various applications that require precise control of fluid flow and interactions. The dimensions of microfluidic channels directly influence fluid behavior, including flow rate, pressure drop, mixing, and diffusion [6]. Any variation in channel dimensions can lead to inconsistent results and unreliable experimental outcomes. The flow rate of a fluid through a microfluidic channel is directly proportional to the channel dimensions, such as width, height, and length [7]. By precisely controlling these dimensions, researchers can manipulate the flow rate and achieve desired fluid velocities [8]. This is crucial for applications such as drug delivery, where precise control of flow rate is necessary to ensure accurate dosing. In microreactors, where chemical reactions occur within microfluidic channels, precise dimensions ensure efficient mixing and reaction rates [9]. Similarly, in biological applications, such as cell culture and analysis, constant channel dimensions allow for consistent fluid flow and controlled interactions between cells and reagents [10]. Compliance is the change in volume for any given applied pressure. When using a syringe pump as the system’s input source, the time it takes for the system to reach steady flow conditions can vary from seconds to hours depending on the fluidic resistance and compliance. This time, between the initial state and when steady flow conditions are reached, is called the response time or the characteristic time.

PDMS has a low elastic modulus (1 to 3 MPa), which allows the deformation of the device under high pressures [3,4,11]. At a low Reynolds number, the law that governs fluid flow is the Hagen Poiseuille model. The hydraulic resistance of a microchannel is inversely proportional to the hydraulic diameter of the channel raised to the power of four (4) [12]. As a result, a small variation in this dimension, due to the deformation of the elastomer resulting from pressure, can cause significant variation in the hydraulic resistance, and thus invalidate the expected flow rate. Additionally, the accurate evaluation of channel dimensions is of utmost significance, as it significantly impacts shear estimation. In microrheology studies, this can directly influence the formulation of viscosity laws and the establishment of relationships between shear and microstructures [13,14,15].

To overcome the limitations associated with PDMS, other materials have been used for the fabrication of microfluidic devices. One of the promising materials is the optical glue Norland Optical Adhesive (NOA). The manufacturing process used to design the NOA chip is comparable to that of the PDMS gold standard [16,17,18]. This material is clear and has a high Young modulus (1655 MPa) [17]. This allows NOA to be used in high-pressure flow systems with minimal compliance as shown by Elodie Sollier et al. when comparing PDMS to other forms of polymer-based materials, including NOA, in high-pressure flow systems [19]. Their findings revealed that the maximum pressure (Pmax) at which delamination occurs in NOA is approximately 74–79 PSI, while PDMS bonded to glass experienced delamination at pressures as low as 36 PSI [19]. This study compares the compliance of PDMS microfluidic devices to that of NOA microfluidic devices, while also assessing the material roughness and hydrophilicity. Surface roughness increases the surface area, which leads to absorption effect and the possibility of trapped air bubbles during flow [20]. So, it is important to study the surface roughness of both materials due to the challenge of micro-scale control, the interfacial properties, the complex boundary effects and the lack of theoretical characterization [20]. Hydrophilicity is vital in microfluidics for the precise handling of small liquid volumes. In a “blood-on-a-chip” device, hydrophilic surfaces enable the accurate manipulation of blood samples within microchannels, facilitating precise diagnostic and analytical procedures. Indeed, hydrophilicity is vital in microfluidics for the precise handling of small liquid volumes. In a “blood-on-a-chip” device, hydrophilic surfaces enable the accurate manipulation of blood samples within microchannels, facilitating precise diagnostic and analytical procedures [21].

## 2. Materials and Methods

### 2.1. Microfluidic Devices Geometry

**Figure 1:**
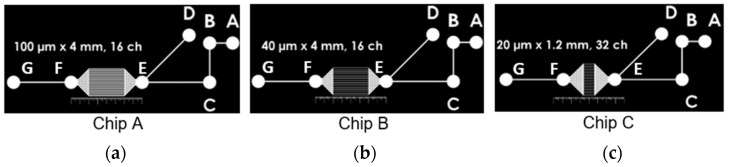
Mask used for SU-8 structures to make the PDMS stamp of (**a**) chip A, (**b**) chip B and (**c**) chip C. The parallel channel widths are 100 μm for chip A, 40 μm for chip B and 20 μm for chip C.

The geometries of the microfluidic chips used were previously designed in Niko Lee-Yow’s study and consist of two tapered chambers connected with parallel channels (Figure 1) [22]. The chip has an inlet path and two outlet paths. Three chips (A, B and C) of different dimensions were used. The dimensions of the parallel channels, tapered chambers, and inlet and outlet paths for the different chips are presented in Table 1 and Table 2. Three chips were designed to present the same order of total resistance.

### 2.2. Manufacturing

NOA63 and NOA81 were both considered, as they were both used in previous studies as microfluidic device materials [17,18]. NOA63 was chosen due to its increased viscosity and low shrinkage [17,18].

#### 2.2.1. SU-8 Wafer

The molds used for the replica molding of PDMS chips were made with SU-8 photoresist patterned on 3-inch silicon wafer. After Piranha solution cleaning (mixture of H_2_SO_4_ and H_2_O_2_) and nitrogen drying, a thin layer of SU-8 50 (MicroChem, Westborough, USA) was manually poured onto the surface of the silicon wafer and spin coated at 500 rpm with an acceleration of 100 rpm/s for 10 s [23]. Immediately after the spread step, a final speed of 1000 rpm was achieved at an acceleration of 300 rpm/s and held for a total of 30 s. After two-step soft baking at 65 °C for 10 min and 95 °C for 30 min, a thin layer of glycerol was used between the mask and wafer to improve the contact [23]. Glycerol was used to ensure a similar refractive index so the light will not refract in the gap between the wafer and mask [24]. The mask/glycerol/wafer was exposed to UV at 500–650 mJ/cm^2^ at 350–400 nm [23]. The mask used for the SU-8 structure imprint is negative, as shown in Figure 1. A post-exposure bake at 65 °C for 1 min and at 95 °C for 10 min was completed on the SU-8 wafer [23]. Once the SU-8 wafer was cooled to room temperature, it was submerged into the SU-8 developer for 10 min, rinsed with isopropyl alcohol (99%) and dried with nitrogen gas.

#### 2.2.2. NOA Device

**Figure 2:**
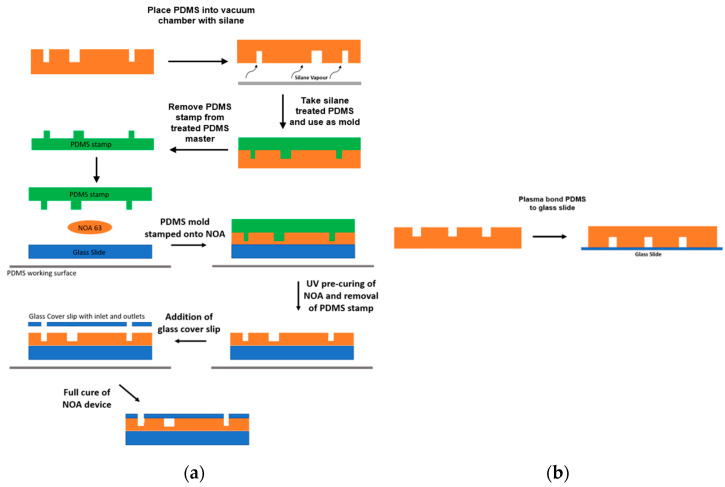
(**a**) Schematic layout of the NOA device manufacturing method using NOA 63. NOA microfluidic chips require a PDMS of the channels to makes a stamp, which is taken from the patterned wafer. A PDMS device is created using a 5:1 ratio of the main agent and the curing agent thus creating a harder PDMS mold [26,27,28]. The mold is treated by vaporizing trichloro (1H, 1H, 2H, 2H-perfluorooctyl) silane (PFOTS) in a vacuum [25]. This leaves a small film of PFOTS on the surface of the PDMS, which can then be used as its own mold to fabricate PDMS devices with correctly oriented channels from the original PDMS device. The PDMS stamp is then slowly placed down on top of the NOA. The NOA is precured using UV light of 365 nm wavelength and 40.25 mW/cm^3^ intensity. The PDMS is then peeled off and the remaining NOA becomes an inverse replica of the PDMS stamp. A glass cover slip with the appropriate inlet and outlet holes is placed on top of the precured NOA chip. The NOA chip is then fully cured for 20 min under UV light. (**b**) Schematic layout of the PDMS device manufacturing method using a PDMS device. The PDMS device is taken from the patterned wafer and plasma bonded to a glass cover slip.

NOA63 was chosen due to its high viscosity (2500 cps) at 25 °C and low shrinkage of approximately 1.5% [16,17]. NOA devices are manufactured using a patterned PDMS stamp. The details of the NOA device manufacturing process are shown in Figure 2a. Using a silanization process, the PDMS molded from the SU-8 wafer was used to fabricate the PDMS stamp. The PDMS-PDMS replica molding was adapted from Zhuang et al. [25]. The PDMS was created using a PDMS 5:1 mixture of main agent and curing agent, respectively. This creates a harder mold allowing the PDMS to act as a more effective mold during the silanization process [26,27,28]. The harder PDMS was placed in a vacuum chamber, directly above 2 drops of trichloro (1H 1H 2H 2H-perfluorooctyl) silane (PFOTS). The PDMS master was de-gassed for 15–20 min causing the PFOTS to create a thin layer over the PDMS surface. The PDMS was then placed on a hot plate for 10 min at 150 °C, before being placed in a pool of deionized water for 10 min to remove any excess silane (PFOTS) that may remain on the PDMS. It could then be used as a mold for the PDMS stamp by using a traditional 10:1 ratio of main agent to curing agent, as typically used in microfluidic devices [28]. The 10:1 ratio was then poured on top of the PDMS and heated at 75 °C in the oven for 1 h. The PDMS stamp was finally cut away from the PDMS.

The manufacturing method of the NOA63 microfluidic devices was adapted from the technique presented by Sim, Jae Hwan et al. [16]. A 1.25 cm diameter circle of NOA63 was dispensed onto a standard glass slide. The PDMS stamp generated from the double molding with a classical SU-8 wafer was used to manually stamp the uncured NOA63. The stamped NOA63 was then placed under 365 nm wavelength UV light, of intensity 40.25 mW/cm^2^, for 10 s causing the glue to stay in a precured state for a total energy exposure of 402.5 mJ/cm^2^. Therefore, the stamped channels were retained by the NOA. The PDMS stamp was removed from the NOA and a glass cover slip was placed, and aligned, on top of the precured NOA. The cover slip was prepared in advance with laser cut holes for the appropriate inlet and outlet ports using a CO_2_ laser etching machine (*Epilog Laser*). The etching machine was set to a speed of 85% and a power of 90%. The coverslip was secured to a wooden surface and 10 laser shots were used to create the inlet and outlet holes. Once aligned atop the precured NOA63, the entire NOA63 device was placed under UV light at an intensity of 40.25 mW/cm^2^ and a wavelength of 365 nm for 20 min for a total energy exposure of 241.5 J/cm^2^. An airtight adhesive bond formed between the glass and the precured NOA. Pieces of PDMS were pre-punched at a 19-gauge diameter and plasma-bonded onto the glass coverslip, completing the NOA device.

#### 2.2.3. PDMS Device

The PDMS devices were manufactured using the SU-8 wafer with a patterned mask as presented in Figure 1. The manufacturing process for the PDMS microfluidic devices is detailed in Figure 2.

The PDMS waqs made by using a traditional 10:1 ratio of main agent to curing agent, typically used in microfluidic devices [28]. This mixture was de-gassed for 1 h until no air bubbles were present. The 10:1 ratio was then poured on top of the SU-8 wafer and heated at 75 °C in the oven for 1 h. The PDMS device was finally cut away from the SU-8 wafer. Inlet and outlet holes were pre-punched at a 19-gauge diameter into the device, and it was plasma-bonded to a glass slide.

### 2.3. Surface Roughness

The surface roughness was measured using the *DektakXT profilometer* (Bruker Corporation, Billerica, MA, USA). A PDMS sample of 34 mm length, width of 14 mm and thickness of 2 mm was used. A sample of NOA 63 of 30 mm length, 14 mm width and thickness of 2 mm was used. The PDMS samples were made on a clean SU-8 wafer to assure the surface did not have imperfections. The NOA sample was made on the PDMS surface in contact with the wafer. Three tests were performed for each sample. For the NOA, the tests were performed on a length of 20 mm with leveling. The PDMS tests were performed on a length of 25 mm with no leveling.

### 2.4. Tensile Strength Test

The tensile strengths of the PDMS and NOA63 was measured using the *Instron 4482 materials testing machine* (Instron Corporation, Norwood, MA, USA). Three PDMS samples measuring 74 mm in length, 26 mm in width and 3 mm in height were tested with tensile grips of 10 N. Similarly, three NOA63 samples of 65 mm length, 20 mm in width and 1.6 mm in height were tested using tensile grips of 10 kN. All samples were tested at a rate of 15 mm/min until failure.

### 2.5. Channels Width Deformation

The channels’ deformation under flow conditions was measured to better understand the compliance in the chips. For this purpose, images of the channels were captured using a microscope featuring 40× objective while maintaining a constant flow. Deionized water was pushed from a 500 µL Hamilton syringe by a syringe pump at rates of 25, 50 and 100 µL/h. The channel width was then measured for each flow using ImageJ 1.53.

### 2.6. Contact Angle

The contact angle measurement was used to observe the hydrophilic and hydrophobic properties of the materials. The same samples of PDMS and NOA63 were used for surface roughness measurements. Both PDMS and NOA samples were cleaned with tape to remove any particles. Three tests were performed on each sample with and without plasma treatment. The tests of the samples without the plasma treatment were first performed followed by the tests with plasma treatment. A plastic syringe was utilized to distribute 1 microliter of water on each sample. A digital microscope captured side-view images, and each sample underwent three tests. The contact angle was then measured using ImageJ ROI manager. The plasma treatment was made using the *Laboratory Corona Treater* (Electro-Technic Products inc., Chicago, IL, USA) device for about 1 min on each sample. This device distributes the plasma using an electric current. Plasma was applied to the surface of the material, causing the chips to become hydrophilic.

### 2.7. Experimental Setup

#### 2.7.1. Pressure Controlled Setup for the Resistance of Microfluidic Devices

**Figure 3:**
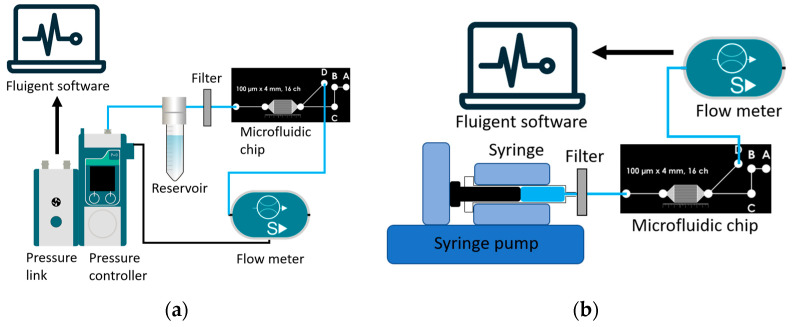
(**a**) Pressure control setup for the resistance testing of the microfluidic devices. Setup includes a pressure controller. All fluid runs through rigid polymer tubing. After exiting the microfluidic chip, the flow is directed into a precision flow meter size S (small). (**b**) Flow set up for the characteristic time testing of microfluidic devices. Setup includes a precision syringe pump with 500 µL glass syringes with 20-gauge metal tubes. All fluid runs through rigid polymer tubing. After exiting the microfluidic chip, the flow is directed into a precision flow meter size S (small).

Resistance can be defined as the slope of the linear relationship between pressure and flow. A pressure-controlled system (Flow EZ, Fluigent, France) coupled with a microflow sensor (Flow Unit S, Fluigent, France) was used to test the resistance for all devices. The fluidic circuit consisted of rigid components with low compliant properties, which include 20-gauge metal tubes and small but rigid polymer tubing. Additionally, a 0.2 µm filter was placed at the top of the reservoir to filter the deionized water to prevent the flow meter from clogging. A detailed flow chart of the experimental setup can be seen in Figure 3a. Pressure was reduced by 0.5 mbar every 20 s from around 100 µL/h until no flow rate was detected. Pressure-flow data were collected with and without the chip and analyzed to calculate the resistance. The process was repeated three times for all three chips for both materials. The average of the flow for each applied pressure was used to plot the pressure flow. The chip’s resistance was then calculated by deducting the resistance of the tubing and filter alone from the resistance of the whole circuit, which included the chip. A total of 6 microfluidic devices were tested: 3 PDMS devices and 3 NOA devices.

#### 2.7.2. Flow Setup for the Characteristic Time of the Microfluidic Devices

A flow-control system was used to test the characteristic time of the devices by analyzing the time delay of the output flow rate versus the flow rate imposed by a pump. The setup consisted of a glass syringe (500 µL *Hamilton*) mounted in a syringe pump (*Nexus 3000*, Chemyx, Stafford, TX, USA) couple with a microflow sensor (Flow unit S, Fluigent, France). The fluidic circuit was composed of 20-gauge metal tubes, and small but rigid polymer tubing. As for the pressure control setup, a filter was placed at the tip of the syringe to filter the deionized water. A detailed flow chart of the experimental setup can be seen in Figure 3b. The syringe pump was set to accommodate flow rates of 25 µL/h, 50 µL/h, and 100 µL/h through the microfluidic device. The microflow sensor data were compared to the syringe pump’s flow rate settings to calculate the characteristic time, in accordance with Section 2.8.3. The process was repeated 3 times and the average results at each point were used to plot flow over time for each chip and flow rate. A total of 6 microfluidic devices were tested: 3 PDMS devices and 3 NOA devices.

### 2.8. Analysis

The fluidic system was modeled using the two-element Resistance Compliance (RC) hydraulic circuit model in Section 2.8.2 [29]. The data were collected from the Fluigent flow software and were processed using MATLAB software. This was done by fitting an exponential function, derived from the RC hydraulic circuit model to the flow rate over time plot as shown in Section 2.8.3.

#### 2.8.1. Theoretical and Hydraulic Resistance Estimation

The hydrodynamic resistance is the slope of the line of the pressure over the flow. The hydrodynamic resistance can be expressed as follows [30]:

(1) $$R_{hyd\;}=\frac{\Delta P}{Q}$$

where ΔP is the difference of pressure, $R_{\mathrm{hyd}}$ is the hydrodynamic resistance of the microfluidic device and Q is the flow.

The theoretical hydrodynamic resistance of the devices is computed by summing the individual resistances in series or parallel as with electrical circuits using the following equations [22]:

(2) $$R_{eq_{serie}}=R_{hyd_{1}}+R_{hyd_{2}}+\cdots +R_{hyd_{n}}$$

(3) $$R_{eq_{parallel}}{}^{-1}=R_{hyd_{1}}{}^{-1}+R_{hyd_{2}}{}^{-1}+\cdots +R_{hyd_{n}}{}^{-1}$$

The hydrodynamic resistance of the rectangular cross-section channels can be calculated using the Hagen–Poiseuille equation as follows [22]:

(4) $$R=\frac{12\;µL}{wh^{3}\left(1-\frac{0.63h}{w}\right)}$$

where *h* is the height of the channel, *w* is the width of the channel, µ is the viscosity of the fluid, and *L* is the length of the channel in question. The hydrodynamic resistance of the tapered channels can be calculated using the Darcy law, as follows [22]:

(5) R=9λπμ28r2hΔr52b1+b2

where *b*_1_ is the width of entrance, *b*_2_ the width of exit, h the height of the channel, r the radius of the micropillar, *λ* the length of the tapered channels and Δ the half distance between fibers.

The experimental resistance of the chip can be obtained by plotting the pressure over the mean flow. The regression line is then modeled as follows:

(6) $$\Delta P=R_{\exp}\times Q+P$$

where $R_{\exp}$, the slope, is the experimental resistance of the microfluidic device, Q is the average flow at each pressure and *P* is the initial pressure. To get the experimental resistance of only the chip, the slope of the regression line with and without the chip must be modeled. Then, the slope of the system without the chip can be subtracted from the slope of the full system (with the chip) as follows:

(7) $$R_{{\mathrm{chip}}_{\exp}}=R_{full\;system}-R_{no\;chip}$$

#### 2.8.2. Two-Element Resistance Compliance (RC) Hydraulic Circuit Model

The flow of the system can be described by:

(8) $$Q_{in}-Q_{out}=Q_{c}$$

where *Q_in_* is the inflow to the system, controlled by the syringe pump. *Q_out_* is the outflow of the system measured by the flow meter, and *Q_c_* is the rate of storage of the system itself. *Q_c_* can be further described by:

(9) $$Q_{c}=C\frac{dP}{dt}$$

where *C* is the compliance of the system and $\frac{dP}{dt}$ is the pressure change over time inside the system. This means the volumetric rate of storage of the system is directly related to the compliance of the system. *Q_out_* can be defined, assuming Hagen–Poiseuille flow, as:

(10) $$Q_{out}=\frac{\Delta P}{R_{s}}$$

where ΔP is the drop-in pressure of the system, and R_s_ is the peripheral resistance. When the pressure at the outflow is assumed to be close to zero, it is reduced to the pressure within the storage chamber. Thus Equations (8)–(10) can be re-written as:

(11) $$Q_{in}-\frac{p}{R_{s}}=C\frac{dp}{dt}$$

Integrating to solve for $p\left(t\right)$ using initial conditions of $p=p_{o}$ (initial pressure) and t=0 (time) the pressure can be written as:

(12) $$p\left(t\right)=R_{s}Q_{in}-\left(R_{s}Q_{in}-p_{0}\right)e^{-\left(\frac{t}{R_{s}C}\right)}$$

where $p_{0}$ is the initial pressure and t is the time. From Equation (12), dp/dt can be rewritten as:

(13) $$\frac{dp}{dt}=\left(\frac{R_{s}Q_{in}-p_{0}}{R_{s}C}\;\right)e^{-\left(\frac{t}{R_{sC}}\right)}$$

Combining Equations (13), (9) and (8), the system equation can be rewritten as:

(14) $$Q_{out}=Q_{in}-\left(\frac{R_{s}Q_{in}-p_{0}}{R_{s}}\right)e^{\left(-\frac{t}{R_{s}C}\right)}$$

where *p*_o_ is the initial pressure of the system, t is the time, and *R_s_C* represents the characteristic time of the system.

#### 2.8.3. Characteristic Time and Compliance

The characteristic time of each trial is calculated and used to compare the compliance of the system. This is assuming that the resistance of the external system is constant and the only change in compliance from the system comes from the microfluidic device.

The outflow of the system, $Q_{out}$ (which is a function of time *t*), can then be modeled using the following equation:

(15) $$Q_{out}=A-Be^{-(\frac{t}{D})}$$

The three constants are used to fit the raw data, acquired experimentally, from Equation (14). The constant *A* represents the *Q_in_*, the constant *B* represents the maximum rate of storage, and the constant *D* is the characteristic time of the system.

With the characteristic time, the compliance can be calculated using the following equation:

(16) $$C=\frac{\tau }{R_{e}}$$

where *C* is compliance, τ is the characteristic time experimentally found for PDMS or NOA using Equations (8)–(15), and *R_e_* is the total experimental resistance of the microfluidic device found using the pressure controller.

### 2.9. Statistical Analysis

Unpaired Student’s *t* test was used for comparisons between the two groups. GraphPad Prism 9 was used to perform the statistical tests and the graphical representations. A *p* value less than 0.05 was considered to be statistically significant. The data were considered a normal distribution, due to the nature of the data describing a physical property of a material [31].

## 3. Results

### 3.1. Mechanical Properties

The surface roughness of NOA63 and PDMS was found in terms of the arithmetic mean height of primary profile (Pa) and the root mean square height of the primary profile (Pq). The results are presented in Table 3. The samples of NOA63 and PDMS underwent three tensile strength tests each with the Instron machine. The average Young’s modulus found for each material is compared to the theoretical values in Table 3.

It was observed that NOA63 has an arithmetic mean height of the primary profile and root mean square height about 10.6 times higher and 11.0 times higher than the PDMS, respectively.

### 3.2. Channels Width Deformation

**Figure 4:**
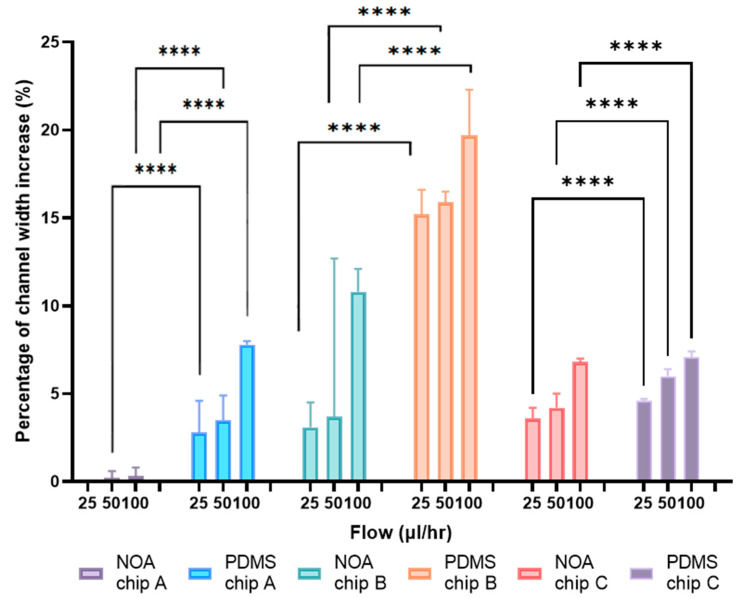
Comparison of NOA and PDMS channels’ width deformation of chip A, B and C at the different flow rates (25, 50 and at 100 µL/h). The initial channel width of the PDMS chip A is 95.4 µm, that of chip B is 36 µm and that of chip C is 17.7 µm. The initial channel width of NOA chip A is 101.7 µm, chip B is 38.2 µm and chip C is 25.6 µm. (**** *p*-value < 0.0001).

The channels of the NOA63 and PDMS microfluidic devices were measured using microscopy and ImageJ at the different flow rates (25, 50 and 100 µL/h). The average values of these measurements are presented in Figure 4.

It was observed that all PDMS devices have a lower initial channel width than NOA devices: 95.4 µm vs. 101.7 µm, 36.0 µm vs. 38.2 µm and 17.7 µm vs. 25.6 µm for PDMS vs. NOA in chip A, B and C, respectively. For all three chips, an increase in the channel width can be observed for the PDMS device as the flow rate increases. For the NOA devices, chip B and C show an increase in the channel width as the flow increases, but chip A displays a constant channel width. For chip A, at 100 µL/h, there is an increase of 7.8% for the PDMS device and of 0.2% for the NOA device. For chip B, at 100 µL/h, there is an increase of 19.7% for the PDMS and of 10.8% for the NOA device. Chip B has the highest increase in the percentage of channel width. For chip C, at 100 µL/h, there is an increase of 7.1% for the PDMS device and of 6.8% for the NOA device. PDMS shows a higher channel increase as the flow rate increases for chip A, B and C than NOA.

### 3.3. Contact Angle

**Figure 5:**
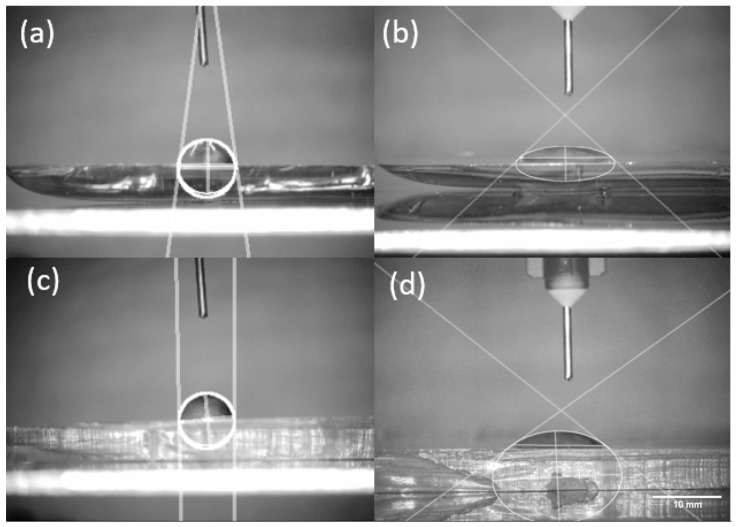
Contact angle for (**a**) NOA63 without plasma treatment (81.4° ± 4.9), (**b**) NOA63 with plasma treatment (44.9° ± 3.1), (**c**) PDMS without plasma treatment (91.4° ± 1.9) and (**d**) PDMS with plasma treatment (32.1° ± 4.4).

The contact angle of the NOA63 and PDMS was obtained with and without portable plasma treatment. Figure 5 illustrates the contact angle of a water droplet on the surfaces.

The results for the contact angle without and with plasma treatment for NOA and PDMS are, respectively, 81.4° ± 4.9, 44.9° ± 3.1, 91.4° ± 1.9 and 32.1° ± 4.4. A higher contact angle means the surface is more hydrophobic, while a lower contact angle means a more hydrophilic surface. By treating the materials with the plasma device, it can be observed that the contact angle decreases for both materials. This means that the microfluidic devices become more hydrophilic after plasma treatment. Without plasma treatment, PDMS is more hydrophobic than NOA, and with plasma treatment, NOA is more hydrophobic than PDMS.

### 3.4. Resistance Estimation

**Figure 6:**
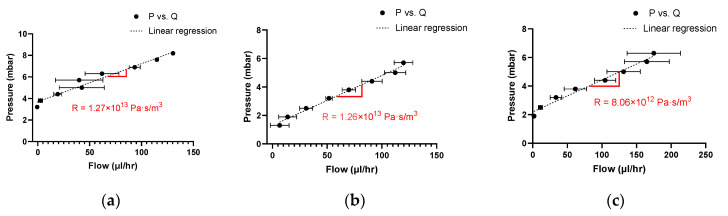
Examples of the pressure in function of the flow for the full hydrodynamic system of (**a**) NOA63 chip A, (**b**) PDMS chip A and (**c**) without the chip.

The hydraulic resistances of the chips were found by following the procedure in 2.7.1 and analyzed using the procedure described in 2.8.1. Examples of the pressure in function of the flow are presented in Figure 6 and the hydraulic resistances are presented in Table 4.

By subtracting the experimental resistance without a chip from the experimental resistance of the NOA63 or PDMS, the experimental resistance of the chip can be found. The experimental resistances of the microfluidic devices are compared to the theoretical values in Table 4.

### 3.5. Characteristic Time Estimation

**Figure 7:**
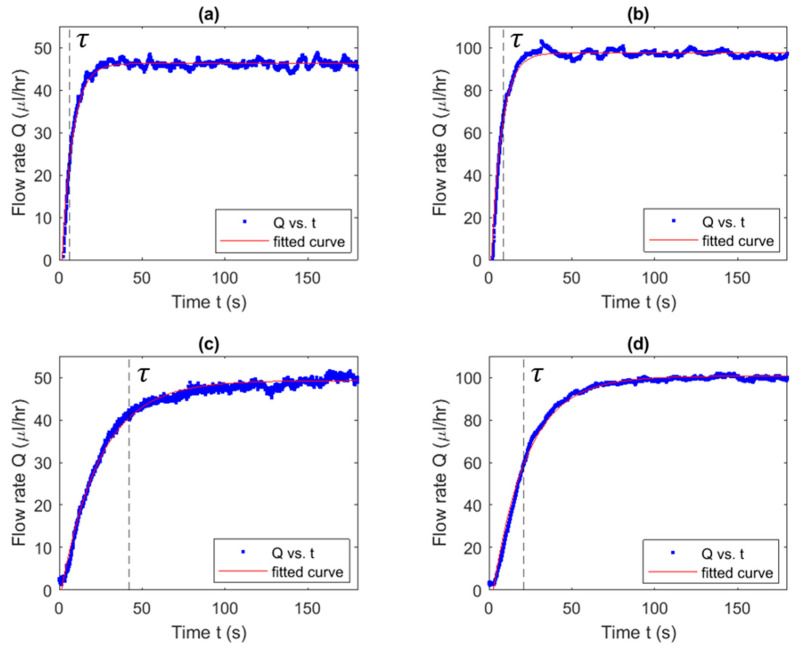
Examples of flow rate versus time graphs for chip A (**a**) at flow rate of 50 µL/h for NOA63, (**b**) at flow rate of 100 µL/h for NOA 63, (**c**) at flow rate of 50 µL/h for PDMS, and (**d**) at flow rate of 100 µL/h for PDMS. Each device shows a significant initial rise from zero to the plateau value (Qout) measured from the Fluigent Flow Meter (S).

Examples of the flow rate as a function of the time for both NOA and PDMS chip A are presented in Figure 7.

**Figure 8:**
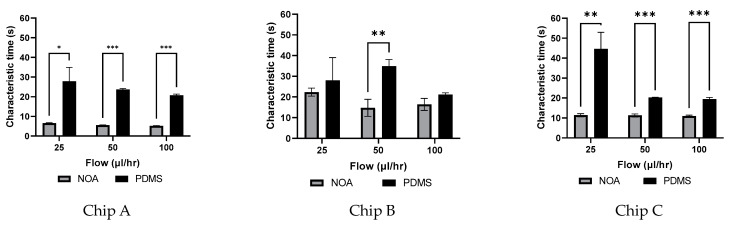
Comparing NOA and PDMS devices of chip A, B and C, average characteristic times at three different flow rates (25, 50, 100 in µL/h) of chip A (* *p*-value < 0.05, ** *p*-value < 0.01, *** *p*-value < 0.001).

All the PDMS and NOA63 trials’ characteristic times were averaged and are graphed in Figure 8.

As shown in Figure 8, there is a significant difference between the characteristic times of the NOA device versus the PDMS device for all chips. For chip A, it was found that the PDMS devices had a characteristic time around 4 times longer than that of the NOA devices. At a flow rate of 25 µL/h, the PDMS device exhibited significantly longer characteristic times compared to the NOA device, which recorded times of 32.2 s and 7.7 s, respectively. For chip B, a significant difference was observed at a flow rate of 50 µL/h. At this flow rate, PDMS had a higher characteristic time than NOA of 34.9 s and 14.8 s, respectively. For chip C, a significant difference can be observed between PDMS and NOA devices at all flow rates. At a flow rate of 25 µL/h, PDMS had a higher characteristic time than NOA, of 44.7 s and 11.4 s, respectively. PDMS has on average a higher characteristic time than NOA devices of 4 times longer for chip A, 1.6 times longer for chip B and 2.5 times longer for chip C. The results presented in Figure 8 suggest a decay of characteristic time as the flow rates increased for all chips.

The statistical analysis revealed that the NOA devices provided more consistent results than the PDMS devices. Chip A showed standard deviations of ±0.38 µL/h, ±0.27 µL/h, and ±0.14 µL/h for flow rates of 25 to 100 µL/h. In comparison, the PDMS devices showed more inconsistency, showing standard deviations of ±12.2 µL/h, ±1.0 µL/h, and ±1.2 µL/h for the same flow rates of 25 to 100 µL/h. Similar trends can be observed for chips B and C.

### 3.6. Compliance

**Figure 9:**
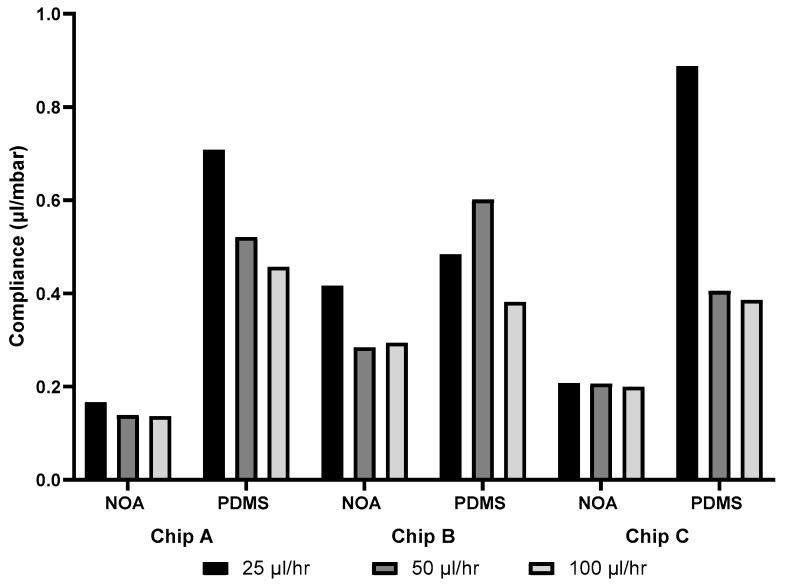
Compliance from characteristic time of both NOA and PDMS chips A, B and C at the different flow rates (25, 50 and 100 µL/h).

The compliance of all devices was found using the time characteristic extracted from Figure 7 and the experimental resistance from Table 4. The compliance found for each device at the different flow rates is presented in Figure 9.

For both PDMS and NOA devices, chip A and chip C showed a decrease in the compliance as the flow rate increased. For chip A, PDMS showed a decrease in the compliance of 36%, while NOA showed a decrease of 18% from 25 µL/h to 100 µL/h. For chip C, PDMS showed a decrease in the compliance of 57%, while NOA underwent a decrease of 4% from 25 µL/h to 100 µL/h. For chip B, a decrease in the compliance could be observed as the flow rate increased for both NOA and PDMS devices, but at some flow rates there was an increase in the compliance.

**Figure 10:**
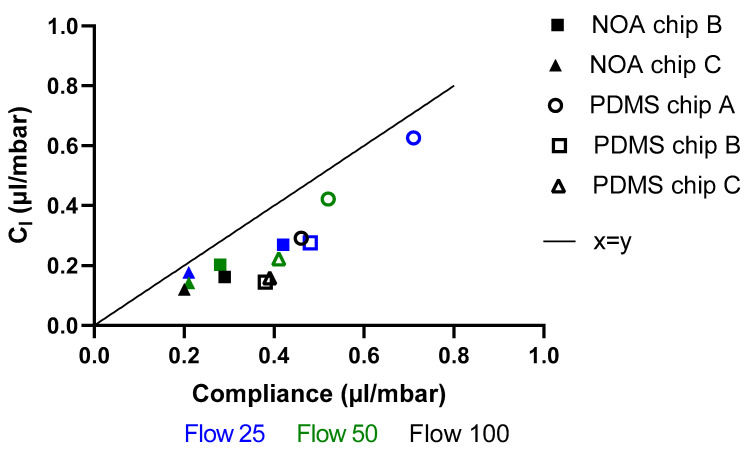
Compliance from the channel deformation in function of the compliance from the characteristic time (*τ*) for all chips at flow rates 25, 50 and 100 µL/h.

An estimation of the compliance using the dimension of the channels can also be given as $C_{l=}\frac{V_{in}\cdot a^{2}}{P}$, where $V_{in}$ is the initial volume, a is the percentage by which the width increased, and P is the pressure applied to flow in the channel. The relation of the compliance calculated from the volume in function of the compliance found from the characteristic time is presented in Figure 10. All experimental outliers were removed.

From Figure 10, it can be observed that for all flow rates there is a correlation between the compliance from the channels deformation and the compliance from the characteristic time, while $C_{l}$ seems underestimated.

## 4. Discussion

### 4.1. Material Properties

#### 4.1.1. Surface Roughness and Young’s Modulus

The surface roughness can be found in Table 4. The mean arithmetic height and root mean square height are about 11 times higher for NOA63 than PDMS. This means that NOA63 has a rougher surface than PDMS. The Young’s modulus of the materials was found in Table 4. The PDMS has a Young’s modulus of 2.1 ± 0.2 MPa and the NOA63 has a Young’s modulus of 1743 ± 173 MPa. These values agree with those in the literature [3,4,11,17]. NOA63 is more rigid than PDMS, which will allow less deformation of the channels under a constant flow, while it is a well-established fact that preparing PDMS with a 1:5 ratio increases its stiffness, resulting in a Young’s modulus of approximately 2.7 MPa [33]. This value remains significantly inferior to that of NOA, which is 800 times higher. This will lead to less compliance in the microfluidic devices. If a different type of Norland Optical Adhesive is used the Young’s modulus changes considerably (325 MPa for NOA81 compared to 1655 MPa for NOA63) [19].

#### 4.1.2. Hydrophilicity/Hydrophobicity of Surface

In Figure 6, a higher intercept, which is the initial pressure, can be observed for the NOA63 device than the PDMS. A higher initial pressure means that the chip is more hydrophobic. The contact angle measurement determines the hydrophilicity/hydrophobicity of the material surface. Both NOA63 and PDMS microfluidic devices received a plasma treatment. NOA63 presented a higher contact angle with plasma treatment (44.9° ± 3.1) and had a higher initial pressure (3.7 mbar) than the PDMS (32.1° ± 4.4 and 1.3 mbar). Therefore, NOA63 microfluidic devices are more hydrophobic than the PDMS.

### 4.2. Experimental Resistance

#### 4.2.1. Channels Width Deformation

A difference between the channel widths of NOA63 and PDMS was observed in Figure 4. This difference can be explained by the double molding. The double molding decreases the average dimension, which explains the narrower width of the PDMS than the NOA [34].

Also, the difference between the resistance values of PDMS and NOA63 for each chip can be explained by the channel’s width deformation. The propagation of error of the channel width was calculated to understand the effect of a difference in the channel’s dimensions. Using a precision error of 0.5% for the width of the channels, the propagation error for chip A can be estimated at 1.24 × 10^13^ Pa·s/m^3^. Due to the channel’s deformation measurement and error propagation, high sensitivity is achieved in the channel’s width measurement for the calculation of the theoretical resistance. This can explain the difference between the experimental and theoretical values in Table 4.

#### 4.2.2. Dependency of Characteristic Time on Flow Rate

The rate of storage for the system, Q_c_, is described by Equation (9) as the compliance (C) multiplied by the change in pressure over time $\left(\frac{dP}{dt}\right)$. As the flow rate increases, the system’s pressure increases as well, which ultimately increases the volume of the rate of storage of the system. By decreasing the resistance, the characteristic time (R_e_C) also decreases. PDMS microfluidic devices had a higher characteristic time for all chips and all flow rates compared to the NOA63 devices. Also, a decrease in the characteristic time can observed as the flow rate increases for all chips. This explains the decay, which becomes evident as the flow rate increases.

### 4.3. Flow Rate Measurement Uncertainty

The Fluigent flow meter S, with a range of ±420 µL/h, was used to accommodate the low flow rates used in the protocol. The accuracy of the device was ±5% of the measured value for all flow rates of 25 µL/h and higher, and ±1.26 µL/h for all measurements below 25 µL/h. The repeatability of the device was within 0.5% for all measurements taken above 42 µL/h and ±0.21 µL/h below 42 µL/h. Thus, all measurements taken were repeatable within 0.5% of the measured value except those taken at 25 µL/h, which were repeatable within 0.84% and 2.1% of the measured value, respectively. This can explain why there are more peaks at lower flow rates for both NOA and PDMS.

The high standard deviations for the PDMS and NOA devices can be explained by the inaccuracy of the Fluigent flow meter at low flow rates (25 µL/h). Although the standard deviations are consistently high for the PDMS devices, the inconsistency in characteristic time, compared to NOA devices, suggests differences in the PDMS vs. NOA devices. In PDMS devices, small variations in material properties can occur with changes in temperature, holding time, and altered mass ratios of pre-polymer to curing agent [26,35]. Furthermore, the PDMS thickness can affect flow conditions and can display significant bulging or deformation during use [4,14,36]. Slight alterations to the PDMS mechanical properties, through uncontrolled parameters, coupled with the channel’s deformation may also contribute to the large standard deviations with PDMS devices. Consistency with the NOA results suggests a more repeatable system than PDMS equivalents. This increase is expected due to the 2% disparity in the Fluigent flow meter at the aforementioned flow rate.

### 4.4. Compliance

Since the flow is modeled using Equation (11), the characteristic time is calculated as system resistance (R_e_) multiplied by the compliance (C). The system setup used was consistent throughout all trials. Therefore, the same rigid tubing and glass syringes were used throughout the entire experimental process. Thus, the only change to the resistance and compliance of the system can be directly associated with the change in the microfluidic device being tested. Due to the low compliance of the materials, the system outside of the microfluidic device was assumed to be negligible when comparing characteristic time values. It is also important to note that the resistance of the system outside of the microfluidic device (syringes and tubing, etc.) is constant. The value was measured to be 8.06 × 10^12^ Pa·s/m^3^. Thus, the characteristic times seen in Figure 8 are directly related to the compliance (presented in Figure 9) of each microfluidic device, therefore suggesting that the PDMS devices have, on average, a higher compliance than that of the NOA devices. A negative correlation between compliance and flow rate has been observed. This indicates that compliance decreases as the flow rate increases. This phenomenon can be explained by the association of reduced deformability with an increase in channel width.

### 4.5. Anticipated Periodic Instability of the Output Flow

The results presented in Figure 7, show the flow rate change over time of PDMS and NOA, respectively. The data demonstrate a clear rise and plateau, following the derived model presented. For both NOA and PDMS devices, the raw data plateaued with a sinusoidal behaviour. This behaviour was analyzed and found to be, as anticipated, caused by the syringe pump’s (*Nexus 3000*, Chemyx, Stafford, TX, USA) stepper motor [37,38].

## 5. Conclusions

The compliance of three different chips of NOA63 microfluidic devices and PDMS microfluidic devices has been quantified under repeatable flow conditions using a pressure-controlled setup and a flow. On average, the characteristic times for chip A were found to be 4 times longer for PDMS devices than NOA devices, 1.6 times for chip B and 2.5 times for chip C. Also, the experimental resistance of the microfluidic device was found for all three chips of both PDMS and NOA. PDMS has a higher percentage of error in the hydraulic resistance due to a higher channel deformation. This leads to a higher compliance. These results concur with the material properties of both NOA and PDMS, having elastic moduli of 1655 MPa and 2 MPa, respectively. The presented results demonstrate that NOA microfluidic devices are less compliant than PDMS microfluidic devices. It also suggests that NOA microfluidic devices could increase consistency in microfluidic research due to their significantly lower standard deviations. This could potentially encourage NOA devices to become the more prevalent option in microfluidic research and in high-pressure microfluidic flow systems. These findings could be used to better understand the use of NOA as a microfluidic material, as well as its properties of compliance and corresponding benefits to microfluidic research.

## Figures and Tables

**Table 1:** Parallel channels and tapered channels dimensions of different chips.

Parallel Channels			
Parameters	A	B	C
Width ($d_{1}$) (μm)	100	40	20
Height ($d_{2}$) (μm)	100	100	100
Length (L) (μm)	4000	4000	1200
Number of parallel channels	16	16	32
**Tapered Channels**			
Width of entrance ($b_{1}$) (μm)	500	500	500
Width of exit ($b_{2}$) (μm)	3100	3100	3740
Height of channel (h) (μm)	100	100	100
Radius of micropillar (r) (μm)	50	50	50
Length of tapered channels (λ) (μm)	1270	1270	1591
Half distance between fibers (Δ) (μm)	50	50	50

**Table 2:** Inlet and outlet channels dimensions.

Parameters	Inlet Channel (Ports GF)	Outlet Channel (Ports ED)	Outlet Channel (Ports EC)	Outlet Channel (Ports CB)	Outlet Channel (Ports BA)
Width ($d_{1}$) (μm)	100	100	100	100	100
Height ($d_{2}$) (μm)	150	150	150	150	150
Length (L) (μm)	5000	6000	6129	2925	1104

**Table 3:** Arithmetic mean height of primary profile (Pa) and root mean square height of the primary profile (Pq) of the surface samples and experimental Young’s modulus of NOA63 and PDMS, as well as those found in the literature.

Sample	Pa (µm)	Pq (µm)	Experimental Young’s Modulus (MPa)	Literature Young’s Modulus (MPa)
**NOA63**	1559 ± 77	1867 ± 98	1743 ± 173	1655 [32]
**PDMS**	147 ± 4	169 ± 4	2 ± 0.2	1–3 [3,4,6]

**Table 4:** Experimental resistance of all 3 chips of NOA63 and PDMS and the theoretical resistance. The theoretical resistance range is calculated with the initial channel width dimension from 0 and the larger width with deformation.

Chip	Theoretical Resistance (10^12^ Pa·s/m^3^)	NOA63 Experimental Resistance (10^12^ Pa·s/m^3^)	PDMS Experimental Resistance (10^12^ Pa·s/m^3^)
**A**	4.78–4.79	4.64	4.54
**B**	5.34–5.66	5.44	5.65
**C**	5.09–5.75	5.44	5.04

## Data Availability

All data that support the findings of this study are included within the article.
